# Extensive post-transcriptional regulation revealed by integrative transcriptome and proteome analyses in salicylic acid-induced flowering in duckweed (*Lemna gibba*)

**DOI:** 10.3389/fpls.2024.1331949

**Published:** 2024-02-07

**Authors:** Lili Fu, Deguan Tan, Xuepiao Sun, Zehong Ding, Jiaming Zhang

**Affiliations:** ^1^ Institute of Tropical Bioscience and Biotechnology, MOA Key Laboratory of Tropical Crops Biology and Genetic Resources, Hainan Bioenergy Center, Chinese Academy of Tropical Agricultural Sciences, Haikou, China; ^2^ Hainan Academy of Tropical Agricultural Resource, Chinese Academy of Tropical Agricultural Sciences, Haikou, China; ^3^ Sanya Research Institute of Chinese Academy of Tropical Agricultural Sciences, Sanya, China

**Keywords:** flowering, *Lemna gibba*, proteome analysis, post-transcriptional regulation, salicylic acid, transcriptome analysis

## Abstract

Duckweed is an aquatic model plant with tremendous potential in industrial and agricultural applications. Duckweed rarely flowers which significantly hinders the resource collection and heterosis utilization. Salicylic acid (SA) can significantly induce duckweed to flower; however, the underlying regulatory mechanisms remain largely unknown. In this work, transcriptome and proteome were conducted in parallel to examine the expression change of genes and proteins in *Lemna gibba* under SA treatment. A high-quality reference transcriptome was generated using Iso-Seq strategy, yielding 42,281 full-length transcripts. A total of 422, 423, and 417 differentially expressed genes (DEGs), as well as 213, 51, and 92 differentially expressed proteins (DEPs), were identified at flower induction, flower initiation, and flowering stages by ssRNA-seq and iTRAQ methods. Most DEGs and DEPs were only regulated at either the transcriptomic or proteomic level. Additionally, DEPs exhibited low expression correlations with the corresponding mRNAs, suggesting that post-transcriptional regulation plays a pivotal role in SA-induced flowering in *L. gibba*. Specifically, the genes related to photosynthesis, stress, and hormone metabolism were mainly regulated at the mRNA level, those associated with mitochondrial electron transport / ATP synthesis, nucleotide synthesis, and secondary metabolism were regulated at the protein level, while those related to redox metabolism were regulated at the mRNA and/or protein levels. The post-transcriptional regulation of genes relevant to hormone synthesis, transcription factors, and flowering was also extensively analyzed and discussed. This is the first study of integrative transcriptomic and proteomic analyses in duckweed, providing novel insights of post-transcriptional regulation in SA-induced flowering of *L. gibba*.

## 1 Introduction

The Lemnaceae (also called duckweed) is a group of small floating plants with high nutrition and rapid growth, showing promising potential in industrial and agricultural applications. First, duckweed grows fast and has high starch content, and it can be utilized as raw materials to produce bioethanol (Yu et al., 2017; Liu et al., 2018). Second, duckweed is stress-tolerant and can remove heavy metals, phosphorus, and nitrogen from sewage, and it has been used to treat municipal and industrial wastewater (El-Shafai et al., 2007). Third, duckweed is abundant in macro- and micro-elements and high in protein content, thus it has widely been applied as animal feeds (Anderson et al., 2011; Pagliuso et al., 2022). However, duckweed reproduces primarily asexually and rarely flowers in natural or laboratorial environments, significantly hindering the collection, conservation, and utilization of germplasms (Pieterse, 2013; Huang et al., 2016; Fu et al., 2020a).

Hormones play vital roles in regulating the flowering process in duckweed, with salicylic acid (SA) being the most critical hormone. SA can induce flowering in both short-day and long-day plants such as *Lemna paucicostata* and *Lemna gibba* (Cleland and Tanaka, 1979; Tanaka and Cleland, 1980; Fu et al., 2017). Other hormones including auxin, ethylene, abscisic acid (ABA), and jasmonic acid (JA) can interact with SA and also contribute to flower-induction in duckweed (Cleland et al., 1982; Krajncic et al., 2006; Fu et al., 2020a). Additionally, day-length is another important environmental factor that regulates duckweed flowering (Cleland and Tanaka, 1979; Tanaka and Cleland, 1980; Fu et al., 2017). Furthermore, hormones and day-length exhibit a combined effect on flower-induction across duckweed species (Fu et al., 2020a; Fourounjian et al., 2021). These results are mainly derived from physiological and biochemical experiments; however, the crucial genes and the underlying regulatory mechanisms of SA-induced flowering remain elusive in duckweed.

Flowering has been demonstrated to be complexly regulated at the transcriptional and post-transcriptional levels. *FLOWERING LOCUS C* (*FLC*) is a key gene in the regulatory network of flowering in *Arabidopsis*, and its expression is transcriptionally and post-transcriptionally regulated (Qi et al., 2019). In addition, *Arabidopsis* flowering genes *FT*, *AP1*, and *AGL24* are also involved in post-transcriptional regulation (Liu et al., 2007; Qin et al., 2017). Similarly, the rice flowering gene *Hd1* is regulated both transcriptionally and post-transcriptionally during adaptation to high latitudes (Goretti et al., 2017), whereas *Ehd1* and *Ghd7* are regulated at the post-transcriptional level (Zhou et al., 2021). These results provide a valuable hint to study the roles of transcriptional and post-transcriptional regulation in the flowering of duckweed species.

Over the past decades, significant advancements have been made in the large-scale identification of genes and proteins in duckweed. RNA-seq was applied to identify key genes relevant to nutrient starvation (Tao et al., 2013), ABA treatment (Wang et al., 2014), NH_4_^+^ response (Wang et al., 2016), radiation exposure (Van Hoeck et al., 2017), salt treatment (Fu et al., 2019; Fu et al., 2020b), and SA treatment (Fu et al., 2020a). A few iTRAQ studies were also performed to identify crucial proteins associated with uniconazole treatment (Huang et al., 2015), nutrient starvation (Huang et al., 2014), and aluminum stress (Su et al., 2019). To date, however, no studies have been conducted in parallel to examine the expression level of genes and proteins, and thus little information is available regarding the associations (such as post-transcriptional regulation) between the transcriptome and proteome in SA-induced flowering of *L. gibba*.

PacBio Iso-Seq is an effective technology to obtain complete cDNA sequences, which are helpful for transcriptomic and proteomic analyses especially when the reference genome is unavailable (Nudelman et al., 2018). In this work, exogenous SA was applied to *L. gibba* and the samples were gathered at day 0 (D0), D12, D13, and D20, which represented four vital time-points during the periods of SA-induced flowering (Fu et al., 2020a). Subsequently, PacBio Iso-Seq was used to produce full-length transcripts for references, and transcriptome and proteome were conducted to detect differentially expressed genes (DEGs) and differentially expressed proteins (DEPs) involved in flower-induction in *L. gibba*. The roles of DEGs/DEPs regulated commonly or exclusively at the mRNA and protein levels were explored. These findings provide a comprehensive understanding of the DEGs/DEPs affected by SA treatment, and enhance our knowledge of post-transcriptional regulation in SA-induced flowering in duckweed.

## 2 Materials and methods

### 2.1 Plant materials and treatment

The *L. gibba* clone 7741 was collected from the Institute of Tropical Bioscience and Biotechnology, Chinese Academy of Tropical Agricultural Sciences, Hainan, China. As described before (Fu et al., 2017; Fu et al., 2020a), *L. gibba* colonies were cultured in 250 mL flasks containing 100 mL liquid MH medium to which 20 μM SA had been added. The pH of the medium was set to 5.9. The colonies were grown under 16 h light/8 h dark photoperiod at 25 ± 1°C, with photosynthetic active radiation of 40 μmol · m^−2^ · s^−1^. The plants grown in the medium without SA supplement were regarded as the control.

According to our previous study (Fu et al., 2020a), D0, D12, D13, and D20 were four vital time-points during SA-induced flowering. Thus, *L. gibba* samples were gathered with three replications at each of these time-points, and immediately frozen in liquid nitrogen and used for subsequent full-length transcriptome, strand-specific RNA-seq (ssRNA-seq), and iTRAQ analyses.

### 2.2 Full-length transcriptome analysis

The library preparation and full-length transcriptome were performed by the Annoroad Gene Technology Corporation (Beijing, China). Briefly, equal quantities of total RNA from each sample (including D0, D12, D13, and D20) were pooled and then utilized for PacBio library preparation with the SMRTbell Template Prep Kit (Pacific Biosciences, CA, USA). The PacBio library was sequenced on the PacBio sequel machine with non-size-selected cDNAs.

Full-length transcripts were generated by the IsoSeq3 program (https://github.com/PacificBiosciences/IsoSeq3), including four modules of ccs, lima, cluster, and polish. Briefly, the circular consensus sequence (CCS) reads were generated from sub-reads by the ccs module, and subjected to remove primers and unwanted sequences by the lima module. Then, polyA tails and artificial concatemers were removed to generate full-length non-concatemer (FLNC) transcripts, which were clustered together by the cluster module. Finally, a consensus sequence was produced for each clustered transcript by the polish module. The high-quality full-length transcripts obtained were subsequently utilized as references for ssRNA-seq and iTRAQ analysis.

### 2.3 ssRNA-seq library construction and sequencing

The ssRNA-seq library construction and sequencing were carried out by the Annoroad Gene Technology Corporation (Beijing, China). The total RNA concentration and quality were analyzed using Nanodrop ND-2000 spectrophotometer (Thermo Scientific, USA) and Agilent 2100 Bioanalyzer (Agilent, USA). Based on the manufacturer’s instruction, the ssRNA-seq libraries were constructed using Illumina TruSeq™ RNA sample prep Kit (Illumina, CA, USA) with Ribo-Zero Magnetic kit for rRNA depletion. The libraries were analyzed on the Illumina Hiseq-4000 machine to generate paired-end reads with 150-bp in length. The samples were repeated three times.

### 2.4 Identification of differentially expressed genes

As described before (Fu et al., 2020a), gene expression levels were estimated by using the RPKM (Reads Per Kilobase per Million mapped reads) method. Low-quality reads and adapter sequences were processed by FASTX-toolkit software (http://hannonlab.cshl.edu/fastx_toolkit/index.html). Clean reads were aligned to the full-length transcripts for abundance estimation by align_and_estimate_abundance.pl from TRINITY 2.1.1 software (Grabherr et al., 2011). DEGs were detected by DESeq2 (Love et al., 2014) setting |log_2_FC (fold-change)| > 1 and the false discovery rate (FDR) < 0.05.

The DEGs were used as the query to perform BLASTP searches against the *Arabidopsis* FLOR-ID database (Bouche et al., 2016) for the identification of flowering-associated genes. WGCNA software (Langfelder and Horvath, 2008) was utilized to identify gene co-expression networks, which were further analyzed by Cytoscape v3.5 (Su et al., 2014).

### 2.5 Protein preparation and LC−MS/MS analysis

The samples utilized for ssRNA-seq were also applied to proteomic analysis by Shanghai Luming Biological Technology Co., Ltd. The concentration of total protein was examined by the bicin-chonininc acid method. Following trypsin digestion, the peptides were dried using vacuum centrifugation, resuspended in 100 mM triethylammonium bicarbonate buffer, and further processed according to the manufacturer’s recommendation for 8-plex iTRAQ. Each sample was examined in two replicates.

The LC-MS/MS assay was conducted as previously documented (Ding et al., 2019). The peptides were mixed and loaded onto a Zorbax Extend-C18 reversed-phase column (5.0 μm, 150 mm × 2.1 mm) on the 1100 HPLC machine (Agilent, CA, USA). Buffer A (2% ACN) and buffer B (90% ACN) were utilized for the reverse gradient. The solvent gradient program was set as follows: 0-8 min, 98% A; 8-8.01 min, 98%-95% A; 8.01-48 min, 95%-75% A; 48-60 min, 75%-60% A; 60-60.01 min, 60%-10% A; 60.01-70 min, 10% A; 70-70.01 min, 10%-98% A; 70.01-75 min, 98% A. The flow rate was set to 300 μl/min.

The mass spectrometer data were obtained by the Triple TOF 5600 machine (SCIEX, USA) which was fitted with a Nanospray III source. Data acquisition was conducted using an ion-spray voltage of 2.4 kV, a curtain gas of 35 PSI, a nebulizer gas of 5 PSI, and an interface heater temperature of 150°C. Survey scans were acquired in 250 ms and up to thirty product ion scans were collected if a threshold of 150 cps with a charge number from two to five was exceeded. The mass range was 350-1500 m/z, and the collision energy was 30 eV.

### 2.6 Identification of differentially expressed proteins

The MS/MS raw files were analyzed for protein identification by Proteome Discoverer v1.3 (Thermo Company, USA) against the *L. gibba* protein database derived from the full-length transcriptome. Credible proteins were identified using the parameters of unique peptides ≥1 and Score Sequest HT > 0 with the blank value removed. The DEPs were identified by the Student’s t-test setting p-value < 0.05 and fold-change < 0.83 (or > 1.2) (Ding et al., 2020).

### 2.7 Integrative transcriptome and proteome analyses

To explore the function of DEGs and DEPs, *L. gibba* genes were annotated and categorized into hierarchical groups based on the MapMan system (Thimm et al., 2004). Significantly enriched functional categories were examined by Fisher’s exact test (Ding et al., 2016; Fu et al., 2016). Correlation coefficients between DEPs and the corresponding mRNAs were computed by the Pearson correlation test.

### 2.8 qRT-PCR assay

As described before (Fu et al., 2020a), total RNA was extracted from the samples using the RNA-prep Pure Plant Kit (TIANGEN Biotech, China). The first-strand of cDNA was generated through reverse transcription using the PrimeScript RT reagent Kit with gDNA Eraser (TaKaRa, Dalian, China). To validate the gene expression levels obtained from ssRNA-seq, fifteen genes related to flowering, hormone, redox, and transcription factor were examined by qRT-PCR method using SYBR-green (TaKaRa, Dalian, China). The qRT-PCR reactions were carried out in triplicate on the Stratagene Mx3005P machine (Stratagene, CA, USA) with the following parameters: 95°C for 45 sec, followed by 40 amplification cycles of 95°C for 10 sec, 60°C for 20 sec, and 72°C for 20 sec. The actin gene was utilized as a reference control (**Supplementary Table S1**), and the relative gene expression levels were determined using the 2^−ΔΔCt^ method (Fu et al., 2020a).

## 3 Results

### 3.1 Full-length transcriptome of *L. gibba*

**Figure 1:**
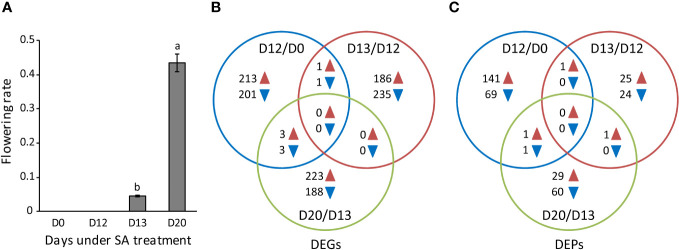
The flowering rate and the identification of DEGs and DEPs. **(A)** The flowering rate of *L. gibba* at D0, D12, D13, and D20 under SA treatment. Data are mean ± standard deviation (n = 3). Different letters indicate significant differences (*P* < 0.05) by Duncan’s multiple range test. **(B, C)** The number of DEGs and DEPs among the flower induction (D12/D0), flower initiation (D13/D12), and flowering (D20/D13) stages. Blue and brown arrowheads indicate down-regulated and up-regulated DEGs/DEPs, respectively.

Our previous studies showed that *L. gibba* was induced to first flower at D13 upon SA treatment, and the flowering ratio reached its maximum at D20 (Fu et al., 2020a). As extended research, *L. gibba* samples were gathered in this study at D0, D12, D13, and D20 for full-length transcriptome assembly (**Figure 1A**), since there is a lack of reference genome in *L. gibba*.

In total, 52.05 gigabases of sequences were produced using the PacBio Iso-Seq platform. These data were processed into 737,364 circular consensus sequencing reads (**Table 1**). After polyA-tail trimming and concatemer removal, 701,677 full-length non-chimeric reads were generated. By using the IsoSeq3 clustering and polishing steps, 42,281 high-quality and 17 low-quality transcripts were found, with a mean length of 1,405 and 2,066 bp, respectively. Subsequently, the 42,281 high-quality transcripts were utilized as references for integrative transcriptomic and proteomic analyses, to investigate the expression change of genes and proteins at distinct stages including flower induction (D12 *vs*. D0), flower initiation (D13 *vs*. D12), and flowering (D20 *vs*. D13).

**Table 1:** The statistics of the initial and processed PacBio Iso-Seq data from *L. gibba*.

Category	Statistic
Number of polymerase reads	784,812
Number of circular consensus sequencing reads	737,364
Number of full-length non-chimeric reads	701,677
Number of high-quality transcripts	42,281
Mean length of high-quality transcripts (bp)	1,405
Number of low-quality transcripts	17
Mean length of low-quality transcripts (bp)	2,066

### 3.2 RNA-seq profiling of *L. gibba*

In total, 953 million clean reads were generated from twelve libraries (four time-points × three replicates) by ssRNA-seq using the Illumina HiSeq 4000 platform. Of which, ~83.88% were aligned to the full-length transcripts of *L. gibba* to quantify the gene expression levels. The total length of these mapped reads was greater than 120.12 gigabases, representing approximately 168-fold coverage of the *L. gibba* full-length transcripts in each sample.

A threshold of RPKM > 1 was chosen to detect genes expressed among samples, resulting in 29,830 expressed genes for DEGs identification. In total, 1,113 DEGs were detected (**Supplementary Table S2**). Of these, 205, 236, and 191 were down-regulated, whereas 217, 187, and 226 were up-regulated respectively at D12/D0, D13/D12, and D20/D13 (**Figure 1B**). It is worthy to note that most down-regulated or up-regulated DEGs were identified exclusively at D12/D0, D13/D12, or D20/D13 while very a few were detected in common, strongly suggesting that distinct functional pathways were affected at each of these stages.

### 3.3 iTRAQ profiling of *L. gibba*

In total, 512,377 mass spectra were produced for eight *L. gibba* samples (four time-points × two replicates). By discarding low-quality spectra and searching against *L. gibba* proteins, 17,871 unique peptides and 4,363 proteins were detected. By setting the parameters of unique peptide ≥ 1 and score sequest HT > 0, 2,242 non-redundant proteins were identified and quantified and subsequently used for DEPs analysis.

In total, 310 DEPs were identified (**Supplementary Table S2**). Of these, 70, 24, and 61 were down-regulated whereas 143, 27, and 31 were up-regulated respectively at D12/D0, D13/D12, and D20/D13 (**Figure 1C**). However, very few DEPs were identified in common, similar to the observation from ssRNA-seq analysis. These results again support that distinct functional pathways were affected at each stage during SA-induced flowering. In the following, we mainly explored the biological function of DEGs and DEPs at these stages, respectively.

### 3.4 Expression correlation between mRNA and protein profiles

**Figure 2:**
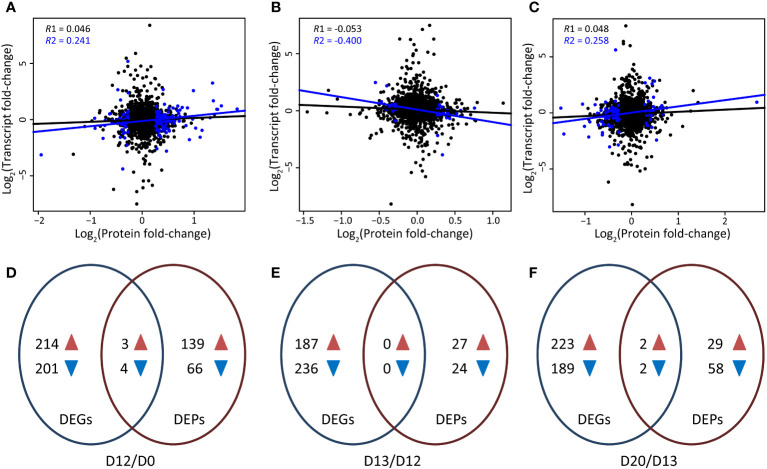
Transcriptomic and proteomic profiling of *L. gibba* at different stages during SA-induced flowering**. (A-C)** Correlation between the expression of proteins and mRNAs at the flower induction (D12/D0), flower initiation (D13/D12), and flowering (D20/D13) stages, respectively. R1 represents the correlation coeficient of all quantified proteins and their corresponding mRNAs, while R2 indicates the correlation coeficient of DEPs and their corresponding mRNAs. Blue and black dots indicate significant and non-significant DEPs, respectively. **(D,–F)** Overlap of DEGs and DEPs at the flower induction (D12/D0), flower initiation (D13/D12), and flowering (D20/D13) stages, respectively. Blue and brown arrowheads indicate down-regulated and up-regulated DEGs/DEPs, respectively.

Correlation analyses were carried out between the transcriptomic and proteomic profiles at D12/D0, D13/D12, and D20/D13, respectively. Of the 2,242 quantified proteins, 1,964 (87.60%) were expressed at the transcriptomic level. Very low correlation coefficients were found at D12/D0 (*R* = 0.046, *P* = 0.054), D13/D12 (*R* = -0.053, *P* = 0.025), and D20/D13 (*R* = 0.048, *P* = 0.041) between all quantified proteins and the corresponding mRNAs. These values were approximately six-fold higher between the DEPs and the corresponding mRNAs at D12/D0 (*R* = 0.241, *P* = 0.002), D13/D12 (*R* = -0.400, *P* = 0.017), and D20/D13 (*R* = 0.258, *P* = 0.022), respectively (**Figures 2A–C**), suggesting a biological relevance of the changes of protein and mRNA profiles during SA-induced flowering in *L. gibba*.

### 3.5 Integrative transcriptomic and proteomic analysis at the flower induction stage

By overlapping the DEGs and DEPs, only three up-regulated and four down-regulated genes were altered with the same trend at D12/D0 at both the mRNA and protein levels (**Supplementary Table S2**). The up-regulated genes were involved in salt stress (*ABH*) and reactive oxygen species (*VAR2*), while those down-regulated genes were referred to ABA response (*CP29B*) and abiotic stress (*RD21*, *APL1*). Then, our main focus was on the DEGs/DEPs that exhibited changes only at the transcriptomic or proteomic level.

**Figure 3:**
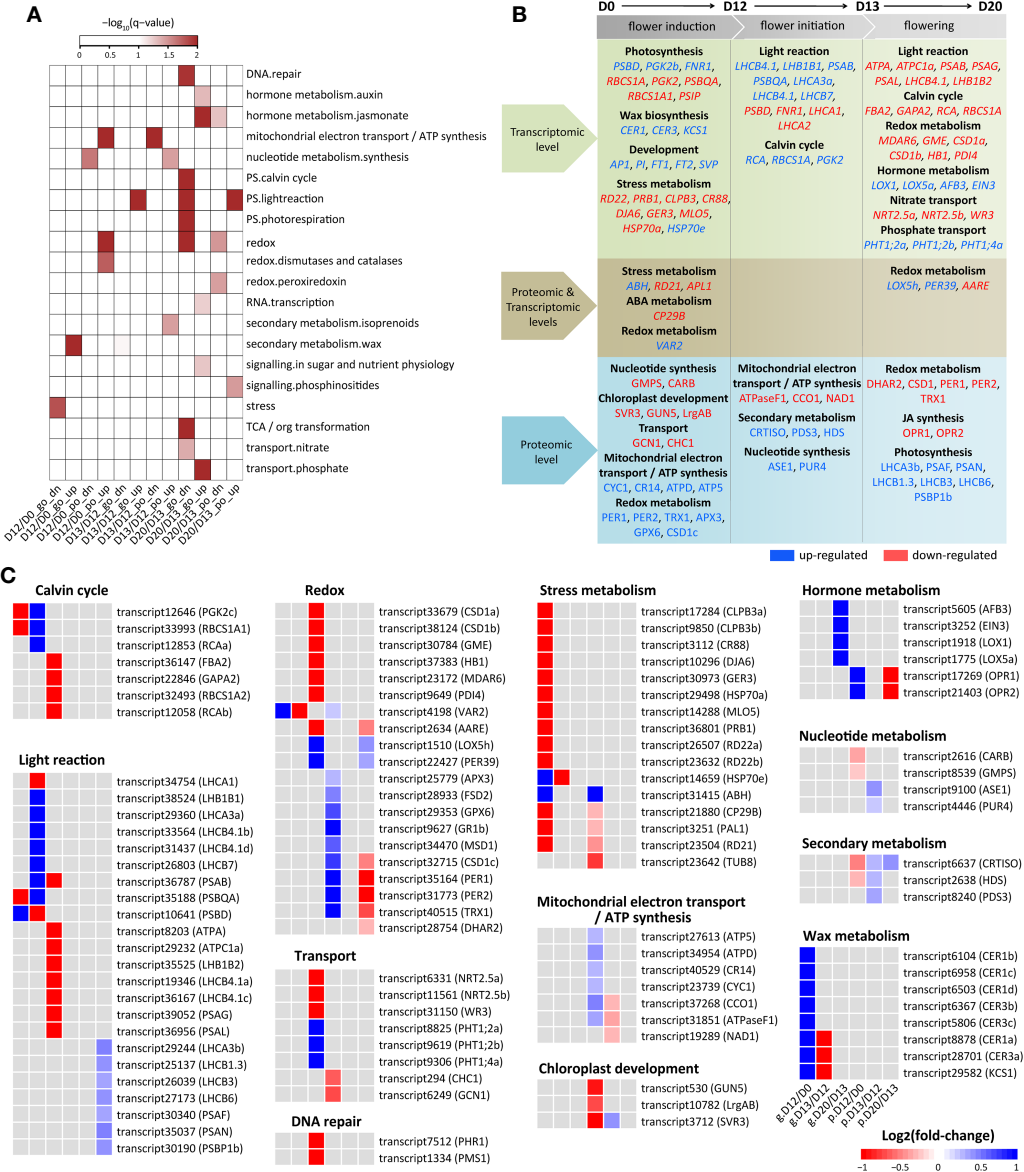
Expression change of DEGs and DEPs involved in SA-induced flowering. **(A)** Functional category enrichment. DEGs/DEPs were classiffied into twelve groups by the integrative transcriptomic and proteomic analysis at each stage. The groups were indicated by stages (D12/D0, D13/D12, and D20/D13) followed by expression level (“go” for gene only and “po” for protein only) and regulatory direction (“dn” for down-regulated and “up” for up-regulated). **(B)** Summary of DEGs/DEPs and pathways influenced during SA-induced flowering. The up- and down-regulated DEGs/DEPs were indicated in blue and red, respectively. **(C)** Heatmap of DEGs/DEPs shown in **(B)**. Rows represent genes with their names in parentheses. The cells from left to right indicate log_2_(fold-change) of genes (prefixed with “g.”) and proteins (prefixed with “p.”) at D12/D0, D13/D12, and D20/D13, respectively.

There were 214 up-regulated and 201 down-regulated DEGs that did not show differential expression at the protein level at D12/D0 (**Figure 2D**). The down-regulated DEGs were enriched in stress metabolism (**Figure 3A**). *RD22* relevant to salt and abscisic acid (ABA), *PRB1* relevant to ethylene, SA, and JA stimulus, *CLPB3*, *CR88*, and *DJA6* related to heat stress, *GER3* related to cold, and *MLO5* related to defense response were significantly down-regulated (**Figures 3B, C**; **Supplementary Table S3**). The up-regulated DEGs were enriched in wax metabolism (**Figure 3A**). Seven genes (including four homologs of *CER1* and three homologs of *CER3*) involved in wax biosynthetic process and cuticle development were significantly up-regulated. *KCS1* (3-ketoacyl-CoA synthase 1), which functions in the elongation of fatty acid during wax biosynthesis, was significantly up-regulated. A few development-related genes were also significantly up-regulated, of which *AP1*, *PI*, *FT1*, *FT2*, and *SVP* played a crucial role in flowering (**Figure 3B**). Notably, four and five of the 10 top most up- and down-regulated genes were involved in photosynthesis (**Supplementary Table S4**), indicating significant expression changes of photosynthesis genes by SA treatment at the flower induction stage.

There were 139 up-regulated and 66 down-regulated DEPs that did not show differential expression at the mRNA level at D12/D0 (**Figure 2D**). These down-regulated DEPs were enriched in synthesis of nucleotide metabolism (**Figure 3A**). GMPS referred to asparagine synthase and CARB referred to arginine synthase were significantly down-regulated (**Figures 3B, C**; **Supplementary Table S3**). Of the 10 top most down-regulated DEPs, several were found to participate in chloroplast development (SVR3, GUN5, LrgAB), transport (GCN1, CHC1), and abiotic stress (TUB8). These up-regulated DEPs were enriched in mitochondrial electron transport/ATP synthesis and redox metabolism (**Figure 3A**). Consistently, a few DEPs involved in electron carrier activity (CYC1), cytochrome c oxidase and reductase (CCO1, CR14), and ATP synthase (ATPD, ATP5) were up-regulated. Many redox-related DEPs, including dehydroascorbate reductase 1 (DHAR1), ascorbate peroxidase 3 (APX3), glutathione-disulfide reductase (GR1), glutathione peroxidase 6 (GPX6), copper/zinc superoxide dismutase 1 (CSD1), Fe superoxide dismutase 2 (FSD2), manganese superoxide dismutase 1 (MSD1), peroxiredoxin 1 (PER1) and PER2, and thioredoxin H-type 1 (TRX1), were also up-regulated. Moreover, PER1, PER2, and TRX1 were included in the 10 top most up-regulated DEPs (**Supplementary Table S4**), indicating a major role of these DEPs in redox metabolism at this stage.

Taken together, these results revealed that numerous genes were significantly changed at the mRNA or protein level: at the mRNA level, stress and photosynthesis related genes were down-regulated while wax and flowering related genes were up-regulated; at the protein level, lots of mitochondrial electron transport/ATP synthesis and redox metabolism related genes were up-regulated, indicating an increase in energy availability for flower induction.

### 3.6 Integrative transcriptomic and proteomic analysis at the flower initiation stage

There were 187 up-regulated and 236 down-regulated DEGs that did not show differential expression at the protein level at D13/D12 (**Figure 2E**). The up-regulated DEGs were enriched in light reaction of photosynthesis (**Figure 3A**). Accordingly, many genes referred to photosystem I subunit (*PSAB*, *PSIP*), photosystem II subunit (*PSBC*, *PSBO2*, *PSBP1*, *PSBQA*), and light harvesting complex (*LHCA3*, *LHCB4.1*, *LHCB7*, *LHB1B1*) were greatly induced. *ATPC1* and *PETA* involved in photosynthetic electron transfer were also induced. Moreover, seven of the 10 top most up-regulated DEGs were involved in calvin cycle (*RCA*, *RBCS1A*, *PGK2*) and light reaction (*LHCB4.1*, *LHB1B1*, *PSAB*, *PSBQA*). Interestingly, six of the 10 top most down-regulated DEGs were also involved in calvin cycle (*PGK2a*, *PGK2b*) and light reaction (*PSBD*, *LHCA1*, *LHCA2*, *FNR1*). These results suggested expression divergence (up-regulated or down-regulated) of photosynthesis genes at this stage.

There were 27 up-regulated and 24 down-regulated DEPs that did not show differential expression at the mRNA level at D13/D12 (**Figure 2E**). The down-regulated DEPs were enriched in mitochondrial electron transport/ATP synthesis (**Figure 3A**). ATPaseF1 encoding a subunit of mitochondrial ATP synthase was significantly down-regulated. Moreover, it was included in the 10 top most down-regulated DEPs (**Supplementary Table S4**). CCO1 and NAD1 involved in mitochondrial electron transport/ATP synthesis were also down-regulated. The up-regulated DEPs were enriched in synthesis of nucleotide metabolism and isoprenoids of secondary metabolism (**Figure 3A**). ASE1 and PUR4 involved in purine biosynthesis were significantly up-regulated. Likely, CRTISO, PDS3, and HDS referred to isoprenoid biosynthetic process were also up-regulated. Moreover, HDS was referred to SA-mediated signaling pathways, establishing a possible connection between secondary metabolism and SA signaling.

However, none of the genes were significantly up-regulated or down-regulated with the same trend at D13/D12 at both the mRNA and protein levels (**Figure 2E**).

Together, these findings suggested that photosynthesis genes were mainly regulated at the mRNA level, while genes relevant to mitochondrial electron transport/ATP synthesis, nucleotide synthesis, and secondary metabolism were mainly regulated at the protein level at the flower initiation stage.

### 3.7 Integrative transcriptomic and proteomic analysis at the flowering stage

There were only two up-regulated and two down-regulated genes dramatically altered at D20/D13 at both the mRNA and protein levels (**Figure 2F**). Three (*LOX5h*, *PER39*, *AARE*) of them were referred to oxidoreductase activity, indicating the involvement of redox metabolism in the flowering of *L. gibba* at the mRNA and protein levels.

There were 223 up-regulated and 189 down-regulated DEGs that did not show differential expression at the protein level at D20/D13 (**Figure 2F**). The enriched categories of the down-regulated DEGs included DNA repair, photosynthesis, redox, and nitrate transport (**Figure 3A**). Many genes participated in calvin cycle (*FBA2*, *GAPA2*, *RCA*, *RBCS1A*), light reaction (including ATP synthases *ATPA* and *ATPC1a*, electron carriers *FEDA* and *DRT112*, photosystem subunits *PSAB*, *PSAG*, *PSAL*, and *PSAO*, and light harvesting complexes *LHCB4.1a*, *LHCB4.1b*, and *LHB1B2*), and photorespiration (*GLDT*, *GOX2*, *HPR*, *PGLP1*) were greatly depressed. Moreover, *PSAB* and *PSB29* involved in light reaction were included in the 10 top most down-regulated DEGs (**Supplementary Table S4**). Consistently, several genes involved in redox metabolism (*MDAR6*, *GME*, *CSD1a*, *CSD1b*, *HB1*, *PDI4*) were depressed. Besides, a few genes involved in DNA repair (*PHR1* and *PMS1*) and nitrate transport (*NRT2.5a*, *NRT2.5b*, *WR3*) were also depressed. The enriched categories of the up-regulated DEGs included hormone metabolism, RNA transcription, sugar signaling, and phosphate transport (**Figure 3A**). *OFT1* and *AFB3* referred to auxin signal transduction, and *LOX1* and *LOX5a* referred to JA synthesis were greatly induced at D20/D13. A nuclear transcription factor (*EIN3*) involved in ethylene response was induced. In addition, several phosphate transporters (*PHT1;2* and *PHT1;4*) were also induced (**Figures 3B, C**; **Supplementary Table S3**).

There were 29 up-regulated and 58 down-regulated DEPs that did not show differential expression at the mRNA level at D20/D13 (**Figure 2F**). The down-regulated DEPs were enriched in JA metabolism and redox (**Figure 3A**). Accordingly, OPR1 and OPR2 involved in JA synthesis were greatly depressed. A few redox-related proteins including DHAR2, CSD1, PER1, PER2, and TRX1 were also depressed. Moreover, PER1 and PER2, and OPR1 and OPR2 were included in the 10 top most down-regulated DEPs (**Figures 3B, C**; **Supplementary Table S3**), indicating their essential roles in SA-induced flowering at D20/D13. These up-regulated DEPs were enriched in light reaction and phosphinositide signaling (**Figure 3A**). Several proteins referred to photosystem I (LHCA3, PSAF, PSAN) and photosystem II (LHCB1.3, LHCB3, LHCB6, PSBP1) were significantly induced. PCAP1a and PCAP1b related to phosphoinositide signaling were also induced. Moreover, LHCB1.3, LHCA3, PSAN, and PCAP1b were included in the 10 top most up-regulated DEPs, indicating their major roles in SA-induced flowering at this stage.

Together, these results suggested that the genes referred to photosynthesis, DNA repair, hormone metabolism, and nitrate and phosphate transport were mainly regulated at the mRNA level, those referred to JA metabolism, light reaction, and phosphoinositide signaling were mainly regulated at the protein level, while those referred to redox metabolism were regulated at the mRNA and/or protein levels at the flowering stage.

### 3.8 DEGs/DEPs related to hormone metabolism

**Figure 4:**
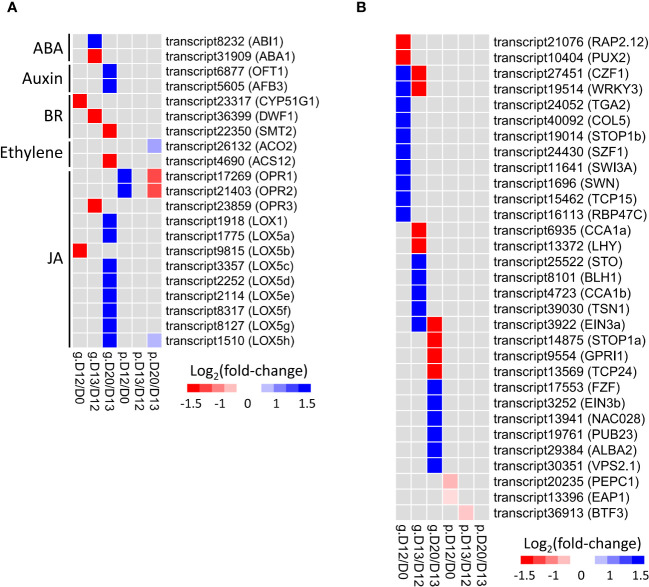
Expression change of hormone **(A)** and TFs **(B)** related DEGs/DEPs involved in SA-induced flowering. Rows represent genes with their names in parentheses. The cells from left to right indicate log_2_(fold-change) of genes (prefixed with “g.”) and proteins (prefixed with “p.”) at D12/D0, D13/D12, and D20/D13, respectively.

In total, 21 DEGs/DEPs related to hormone metabolism were identified by SA treatment at the mRNA and/or protein levels (**Figure 4A**; **Supplementary Table S5**). The most abundant category was JA (12), followed by brassinosteroid (BR, 3), auxin (2), ABA (2), and ethylene (2).

Three oxophytodienoate reductases (*OPR1*-*OPR3*) and nine lipoxygenases (*LOX1*, *LOX5a*-*LOX5h*) referred to JA synthesis were identified. OPR1 and OPR2 were up-regulated at D12/D0 but down-regulated at D20/D13 exclusively at the protein level, while *OPR3* was down-regulated at D13/D12 exclusively at the mRNA level. Interestingly, eight of nine lipoxygenases (except *LOX5b*) were up-regulated at D20/D13 only at the mRNA level.

*CYP51G1*, *DWF1*, and *SMT2* involved in BR synthesis were down-regulated at D12/D0, D13/D12, and D20/D13 exclusively at the mRNA level, respectively. *OFT1* and *AFB3* involved in auxin signaling transduction were up-regulated at D20/D13 only at the mRNA level. *ABA1* and *ABI1* related to ABA synthesis and signaling were down-regulated and up-regulated, respectively, at D13/D12 exclusively at the mRNA level. Two DEGs/DEPs (*ACO2* and *ACS12*) related to ethylene synthesis were identified at D20/D13. The former was up-regulated exclusively at the protein level, while the latter was down-regulated exclusively at the mRNA level.

Collectively, these findings strongly suggested the participation of hormone genes in SA-induced flowering of *L. gibba* via post-transcriptional regulation.

### 3.9 DEGs/DEPs relevant to transcription factors

In total, 28 TF-related DEGs were identified by SA treatment exclusively at the mRNA level (**Figure 4B**; **Supplementary Table S6**). At D12/D0, the down-regulated genes were associated with redox metabolism (*RAP2.12*), while those up-regulated genes were relevant to abiotic stress (*CZF1*, *SZF1*), SA and JA treatment (*TGA2*, *WRKY3*), stamen development (*TCP15*), vernalization (*SWN*), and flowering (*COL5*). At D13/D12, the down-regulated genes were associated with circadian rhythm (*CCA1*, *LHY*), while those up-regulated genes were relevant to ABA and ethylene response (*BLH1*, *EIN3a*), abiotic stress (*STO*, *TSN1*), and embryo sac development (*BLH1*). At D20/D13, the down-regulated genes were associated with chloroplast development (*GPRI1*) and leaf differentiation (*TCP24*), while those up-regulated genes were relevant to abiotic stress (*FZF*, *PUB23*, *ALBA2*) and ethylene response (*EIN3b*).

Only three TF-related DEPs were identified by SA treatment exclusively at the protein level (**Figure 4B**; **Supplementary Table S6**). In contrast, none of the TFs were identified at both the mRNA and protein levels.

Collectively, these findings suggested that TFs played stage-specific roles in SA-induced flowering of *L. gibba* through post-transcriptional regulation.

### 3.10 Identification of flowering-associated DEGs/DEPs and the co-expression network

**Figure 5:**
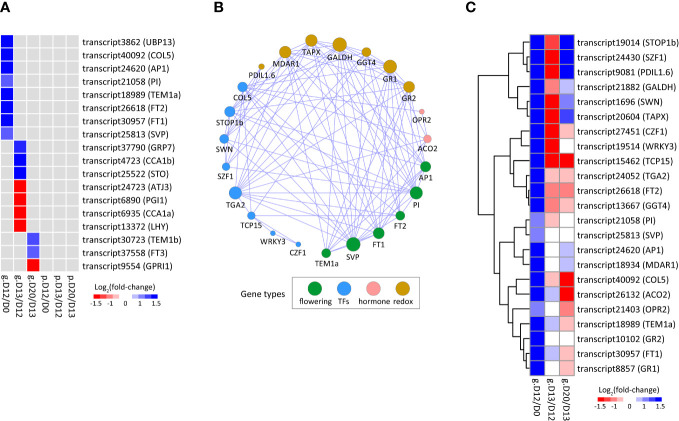
The flowering-associated DEGs/DEPs participating in SA-induced flowering. **(A)** Heatmap of flowering-associated DEGs/DEPs. **(B)** Co-expression network of flowering-associated DEGs. The adjacencies with correlation coefficients more than 0.85 are visualized. The size of a node indicates its connectivity to other genes. **(C)** Heatmap of the DEGs shown in **(B)**. The cells **(A, C)** from left to right indicate log_2_(fold-change) of genes (prefixed with “g.”) and/or proteins (prefixed with “p.”) at D12/D0, D13/D12, and D20/D13, respectively.

A total of eighteen flowering-associated genes exhibited differential expression at the mRNA level but did not show significant changes at the protein level. They were identified at D12/D0, D13/D12, or D20/D13, indicating a stage-specific role of these genes during SA-induced flowering (**Figure 5A**; **Supplementary Table S7**). Notably, the eight DEGs identified at D12/D0 were all significantly up-regulated and they included a few well-known flowering-associated genes (e.g., *AP1*, *FT1*, *FT2*, and *SVP*), thus these genes were used as query genes to conduct co-expression analysis.

The co-expression network contained six flowering-associated genes (**Figures 5B, C**; **Supplementary Table S8**). Of these, *SVP*, *PI*, and *FT1* were the top three hub genes, indicating that they are key players for SA-induced flowering in *L. gibba*. The co-expression network contained eight TFs, of which *SWN* and *COL5* were related to vernalization and flowering, *CZF1* and *SZF1* were related to abiotic stress, and *WRKY3* was related to JA response (**Figures 5B, C**; **Supplementary Table S8**). Two hormone genes were included, including *ACO2* for ethylene synthesis and *OPR2* for JA biosynthesis. In addition, a few redox-related genes were also included, of which *TAPX* and *MD*AR1 functioned in the removal of hydrogen peroxide. Collectively, these findings unveiled a complex regulatory network of genes participating in SA-induced flowering of *L. gibba*.

### 3.11 qRT-PCR verification

**Figure 6:**
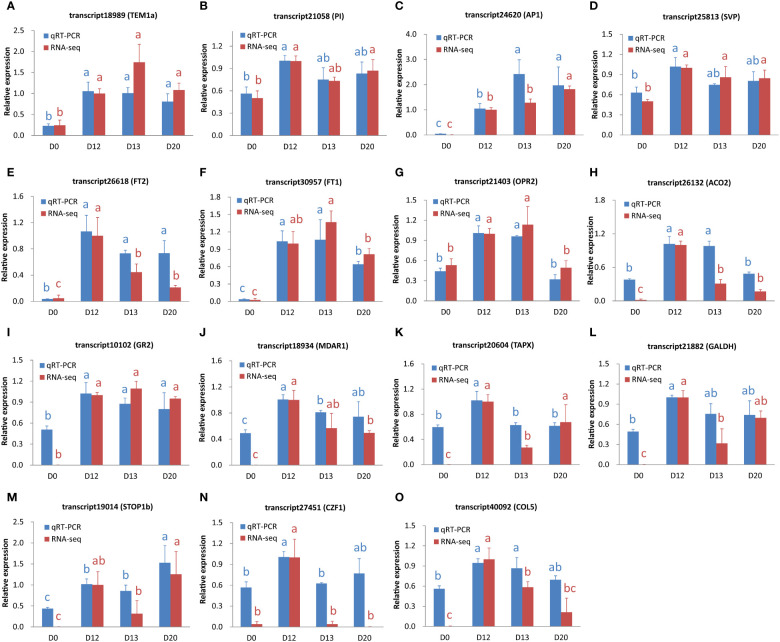
The expression level of genes examined by qRT-PCR and ssRNA-seq. **(A-O)** The expression levels for transcript18989 (TEM1a), transcript21058 (PI), transcript24620 (AP1), transcript25813 (SVP), transcript26618 (FT2), transcript30957 (FT1), transcript21403 (OPR2), transcript26132 (ACO2), transcript10102 (GR2), transcript18934 (MDAR1), transcript20604 (TAPX), transcript21882 (GALDH), transcript19014 (STOP1b), transcript27451 (CZF1), and transcript40092 (COL5), respectively. Data were shown as mean ± standard deviation from three biological replicates. The values indicated by different letters (blue and red for qRT-PCR and ssRNA-seq, respectively) were significant (P < 0.05) according to the Duncan’s multiple range test.

To verify the expression levels of ssRNA-seq data, fifteen genes relevant to flowering, hormone, redox, and TFs were investigated by the qRT-PCR method. The correlation coefficients between the two independent approaches ranged from 0.79 to 0.99 (mean = 0.91, **Figure 6**; **Supplementary Table S1**), suggesting reliable gene expression levels examined by the ssRNA-seq method.

## 4 Discussion

### 4.1 The DEGs/DEPs identified at the transcriptomic and proteomic levels

Transcriptomic and proteomic methods have been widely used to mine critical genes and proteins in duckweed under various treatments over the past decades (Tao et al., 2013; Huang et al., 2014; Huang et al., 2015; Shang et al., 2023). However, no studies are currently conducted by integrated analysis of these two methods, a useful approach to systematically dissect the regulation of complex traits (Ding et al., 2020). To gain a deeper understanding of the molecular mechanism underlying SA-induced flowering of *L. gibba*, in the present study, ssRNA-seq and iTRAQ were simultaneously conducted to identify the DEGs and DEPs at the transcriptomic and proteomic levels.

In total, 1,113 DEGs and 310 DEPs were identified. However, very few of them were commonly detected at the transcriptomic and proteomic levels (**Figures 2D–F**). This suggested that significant expression alterations in proteins did not always correspond to changes at the transcript level. Consistently, low correlations (*R* < 0.40) were observed between the expression level of these DEPs and the corresponding mRNAs (**Figures 2A–C**), in accord with the conclusion of previously reported transcriptome and proteome studies (Lan et al., 2012; Zhang et al., 2016). In particular, the DEPs showed a significant negative correlation with their corresponding mRNAs at D13/D12 (**Figure 2B**). The discordant changes in mRNA/protein levels and the low overlap of DEGs/DEPs strongly suggest a key role of post-transcriptional regulation in SA-induced flowering of *L. gibba*.

### 4.2 Expression regulation of heat shock proteins in flowering of *L. gibba*

Post-transcriptional regulation plays a crucial role in plants under various environmental stresses (Floris et al., 2009), which are now regarded as a new category of factors to regulate flowering in plants (Takeno, 2016). Our previous work has demonstrated that the flowering of *L. gibba* induced by SA treatment is associated with stress responses (Fu et al., 2020a). Therefore, it is of great interest for us to examine the expression regulation of stress-related genes in this current study.

In total, 26 DEGs and six DEPs related to abiotic stress were detected. Of these, 65% (17/26) and 67% (4/6) were heat shock proteins (HSPs) based on the MapMan annotation (**Supplementary Table S9**), suggesting crucial roles of HSPs in flowering of *L. gibba*. The results align with the functions of HSPs in the flowering of *Arabidopsis* (Chen et al., 2019; Qin et al., 2021). The involvement of HSPs in the flowering of *L. gibba* was also confirmed by gene co-expression network analysis, since two homologs of *Hsp101C* (*LPB3a* and *CLPB3b*) showed significant negative correlations with the expression of *SVP* in our work, in accord with the role of *Hsp101* in promoting the flowering of *Arabidopsis* by negatively regulation of *FLC* and *SVP* (Qin et al., 2021). Notably, these HSPs were identified exclusively at the transcriptomic or proteomic level, strongly suggesting the involvement of HSPs in SA-induced flowering through post-transcriptional regulation. These results also established possible molecular connections between flowering and abiotic stresses as recently proposed (Chirivi and Betti, 2023).

### 4.3 Expression regulation of hormone-related genes in flowering of *L. gibba*

Hormones play an essential role in regulating the flowering in duckweed, with SA being the most attractive hormone (Fu et al., 2017; Fourounjian et al., 2021). Interestingly, the SA involved in flower-induction of *L. gibba* was unlikely derived from *in vivo* SA biosynthesis, since the homologs of SA biosynthesis genes from *L. gibba* were either dramatically suppressed by SA treatment or absent in the assembled transcripts (Fu et al., 2020a). Besides SA, other hormones such as ABA, JA, ethylene, and auxin also participate in the induction of flowering in duckweed (Cleland et al., 1982; Krajncic et al., 2006; Fu et al., 2020a). Our previous work identified dozens of hormone-related genes in SA-induced flowering of duckweed; however, it did not examine gene expression at the mRNA and protein levels simultaneously, and therefore it was unable to afford any information regarding the associations between changes in the transcriptome and proteome.

In this work, 18 DEGs and four DEPs related to ABA, auxin, BR, ethylene, and JA were identified, supporting the involvement of these hormones in SA-induced flowering of *L. gibba* (Fu et al., 2020a). Application of ABA showed an inhibitory effect on the flower-induction of *L. gibba* by SA treatment (Fu et al., 2020a). Consistently, we found that *ABA1*, which is responsible for catalyzing the initial step of ABA biosynthesis, showed a significant down-regulation at D13/D12 at the mRNA level (**Figure 4A**). The promoted function of JA in SA-induced flowering of *L. gibba* was supported by the expression up-regulation of many genes associated with JA biosynthesis and signaling pathways (Fu et al., 2020a). Our study confirmed this observation and further found that these JA biosynthesis genes were mainly up-regulated exclusively at the mRNA or protein level. Similarly, *CYP51G1*, *DWF1*, and *SMT2* involved in BR synthesis, *OFT1* and *AFB3* involved in auxin signaling, and ACO2 and ACS12 involved in ethylene synthesis were regulated either at the mRNA or protein level (**Figure 4A**). These discordant changes between the mRNA and protein levels strongly suggest that post-transcriptional regulation plays a vital role in the expression regulation of hormone genes in SA-induced flowering of *L. gibba*, in accord with the roles of post-transcription regulation in hormone metabolism (Farrow, 1993).

### 4.4 Expression regulation of flowering-associated genes in *L. gibba*

Transcriptional regulation and post-transcriptional regulation play a critical role in the flowering of plants (Qin et al., 2017; Zhou et al., 2021). Therefore, elucidating regulatory mechanisms of flowering-associated genes transcriptionally and post-transcriptionally is particularly important for controlling flowering in plants. Our previous study identified 13 flowering-associated genes and proposed a putative regulatory model for SA-induced flowering of *L. gibba* (Fu et al., 2020a). However, it is still unknown whether transcriptional and post-transcriptional regulation participated in the SA-induced flowering of *L. gibba*.

APETALA 1 (*AP1*) is a key gene responsible for floral meristem specification and sepal and petal development (Irish and Sussex, 1990). *AP1* can bind to the promoters of various flowering-time genes, including SUPPRESSOR OF OVEREXPRESSION OF CONSTANS 1 (*SOC1*), SHORT VEGETATIVE PHASE (*SVP*), and AGAMOUS-LIKE 24 (*AGL24*), thereby regulating their expression. Together with PISTILLATA (*PI*), these genes play a redundant role in the early stage of flower development (Gregis et al., 2009). In this work, we found that *AP1*, *PI*, and *SVP* were co-expressed and their expression levels were dramatically up-regulated at D12/D0 only at the mRNA level, suggesting a coordinate role of these genes at the flower induction stage in *L. gibba* via post-transcriptional regulation.

TEMPRANILLO 1 (*TEM1*) and *SVP* act as repressors of FLOWERING LOCUS T (*FT*), a key floral integrator that participated in vernalization, photoperiodic flowering, and stress-induced flowering (Takeno, 2016). Here we found that *SVP*, one homolog of *TEM1* (*TEM1a*), and two homologs of *FT* (*FT1* and *FT2*) from *L. gibba* were co-expressed, suggesting that the interactions of *FT*/*TEM1* and *FT*/*SVP* in *L. gibba* might be different to those reported in other species (Lee et al., 2007; Sawa and Kay, 2011). Interestingly, the expression of *TEM1a*, *FT1*, and *FT2* was also up-regulated at D12/D0 only at the mRNA level, supporting crucial functions of these genes in flowering via post-transcriptional regulation (Qin et al., 2017).

**Figure 7:**
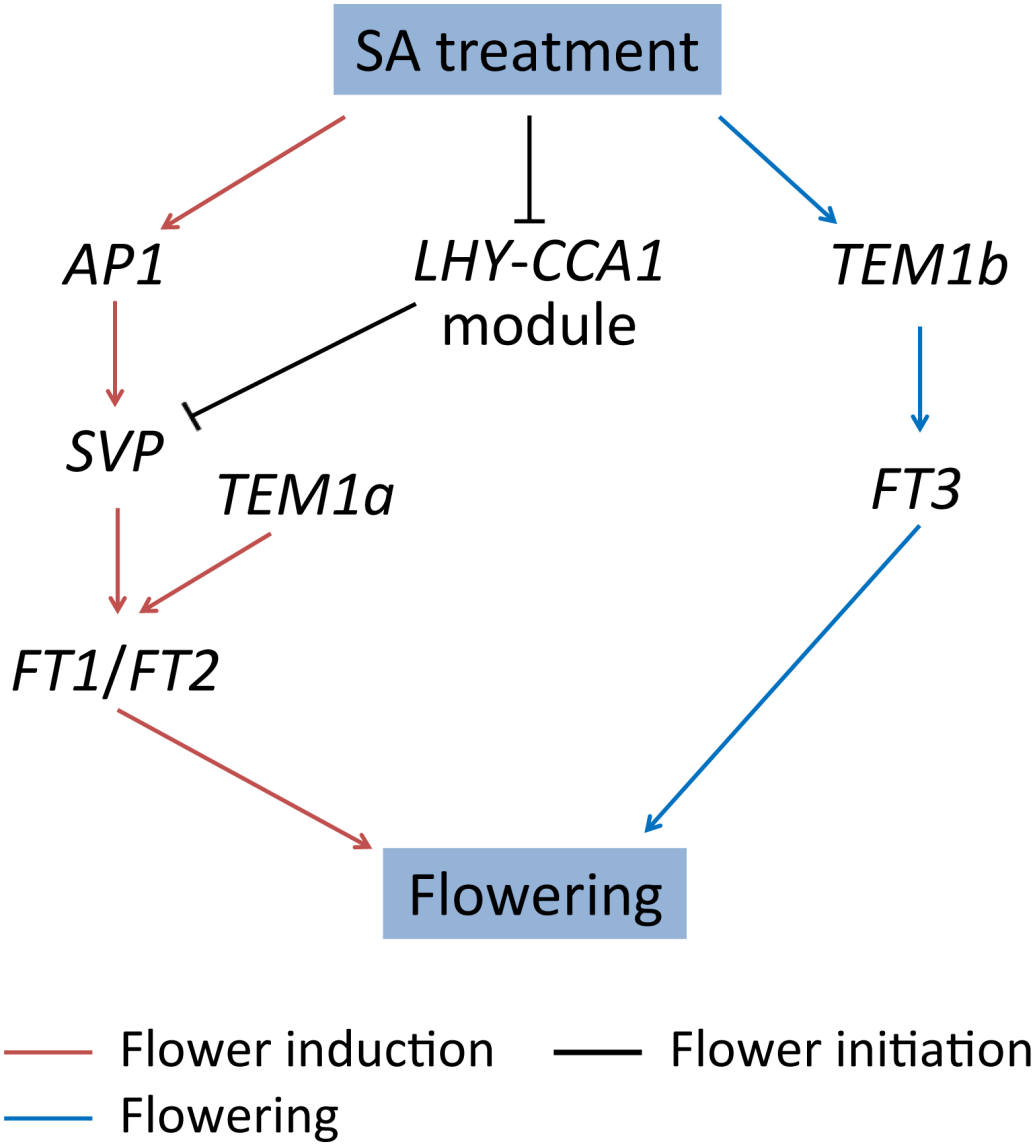
The regulatory models of SA-induced flowering in *L. gibba*. The arrows and T-bars represent positive and negative (inhibitory) regulation relationships, respectively. The regulation relationships at flower induction (D12/D0), flower initiation (D13/D12), and flowering (D20/D13) stages are indicated by brown, black, and blue, respectively.

The post-transcriptional regulation in the flowering of *L. gibba* was also observed at flower initiation and flowering stages. LATE ELONGATED HYPOCOTYL (*LHY*) and CIRCADIAN CLOCK–ASSOCIATED1 (*CCA1*) are crucial clock components that function synergistically in photoperiodic flowering by regulating the flowering-time genes (Fujiwara et al., 2008). In our study, *L. gibba* homologs (*CCA1a* and *LHY*) of these two genes were co-expressed and both down-regulated exclusively at the mRNA level at D13/D12. Likely, *TEM1b* and *FT3* were dramatically up-regulated at D20/D13 only at the mRNA level (**Figure 5A**). The regulatory models of SA-induced flowering in *L. gibba* have been summarized in **Figure 7**. Altogether, the discordant changes of flowering-associated genes in mRNA/protein levels strongly suggested a vital role of post-transcriptional regulation in SA-induced flowering of *L. gibba*.

## 5 Conclusions

In the present work, the transcriptome and proteome analyses were integrated to unveil the regulatory mechanism of SA-induced flowering in *L. gibba*. A total of 422, 423, and 417 DEGs, and 213, 51, and 92 DEPs were respectively identified at the flower induction, flower initiation, and flowering stages. The DEPs exhibited low expression correlations with their corresponding mRNAs, suggesting the crucial roles of post-transcriptional regulation in SA-induced flowering of *L. gibba*. Most DEGs and DEPs were regulated only at the transcriptomic or proteomic level, and their biological roles were systematically studied. The post-transcriptional regulation of transcription factors, hormone genes, and flowering-associated genes was also analyzed and discussed. This is the first study of integrative transcriptomic and proteomic analyses in duckweed, providing novel insights of post-transcriptional regulation in SA-induced flowering of *L. gibba*.

## Data availability statement

The datasets presented in this study can be found in online repositories. The names of the repository/repositories and accession number(s) can be found below: NCBI database (PRJNA631155) and iProX database (IPX0007494000).

## Author contributions

LF: Data curation, Formal analysis, Funding acquisition, Investigation, Validation, Writing – original draft. DT: Investigation, Writing – review & editing. XS: Investigation, Writing – review & editing. ZD: Conceptualization, Formal analysis, Writing – original draft, Writing – review & editing. JZ: Conceptualization, Supervision, Writing – review & editing.
